# Medical First-Trimester Termination of Pregnancy with Massive Fibroid Uterus

**DOI:** 10.1155/2017/8203649

**Published:** 2017-10-02

**Authors:** Michael Saad-Naguib, Karla Maguire, Christine Curry, Usha Verma

**Affiliations:** Department of Obstetrics and Gynecology, Jackson Health System/University of Miami Miller School of Medicine, 1611 NW 12th Avenue, Miami, FL, USA

## Abstract

First-trimester termination of pregnancy by medical or surgical route is highly effective with a low complication rate. Uterine abnormalities can complicate a procedure due to distortion of normal anatomy. In this case presentation, medical termination of pregnancy is performed using fetal intracardiac potassium chloride injection and intramuscular methotrexate.

## 1. Introduction

First-trimester termination of pregnancy by medication or uterine aspiration is highly effective and has a low complication rate [1]. However, there are situations in which uterine anomalies such as a bicornuate uterus or uterine leiomyomata can complicate a first-trimester abortion due to the distortion of normal anatomy [2].

## 2. Case Report

A 38-year-old G4P3003 with no significant past medical history presented at 9 weeks and 5 days of gestation, confirmed by ultrasound. Her pregnancy history included 1 full-term vaginal delivery and 2 full-term cesarean sections. She was previously told by a community OB/GYN to avoid pregnancy as her fibroids placed her at risk of complications including spontaneous abortion, hemorrhage, and maternal death. The patient discontinued oral contraceptives at the recommendation of her OB/GYN due to concern that hormonal contraceptives caused the fibroids to grow. She became pregnant soon thereafter.

**Figure 1:**
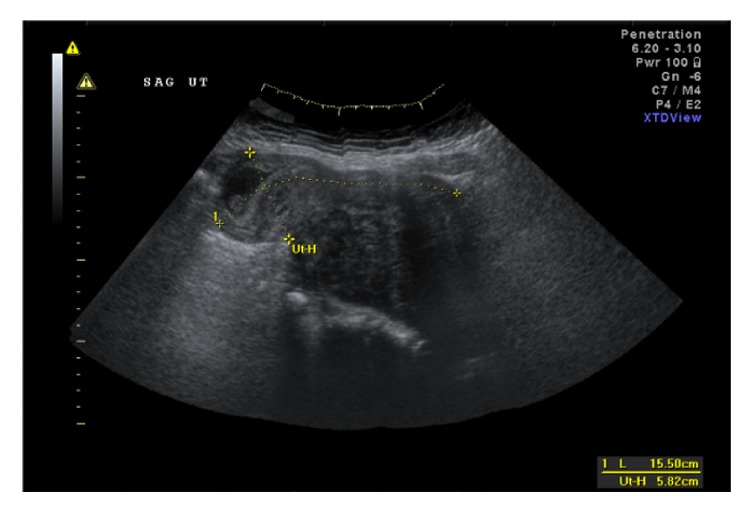
Ultrasound (US) image of fibroid uterus with intrauterine pregnancy.

**Figure 2:**
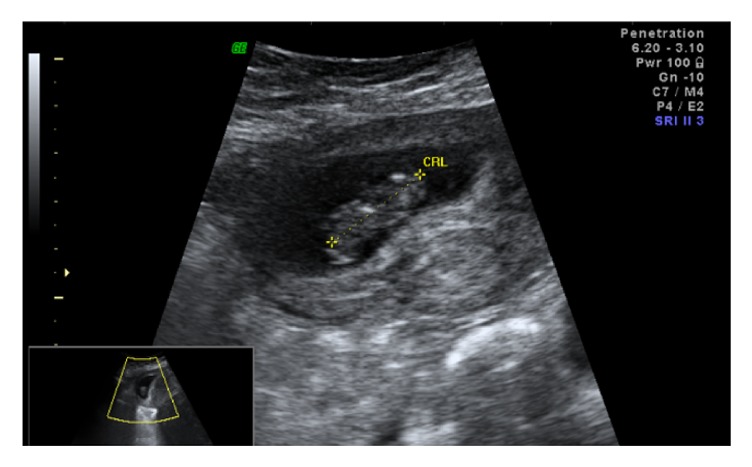
US image of intrauterine pregnancy.

The patient was referred to an outside abortion clinic and was told that a termination was of high risk due to the inability to access the displaced cervix from the vagina. She was then referred to our tertiary care setting. On exam, she had a large fibroid uterus that filled her pelvis, and her cervix was unable to be visualized or palpated. Ultrasound demonstrated an 18.1 × 9.0 × 15.7 cm subserosal fibroid in the posterior uterine wall and a 9-week fetus with cardiac activity at the superior aspect of the uterus (cf. Figures 1 and 2).

**Figure 3:**
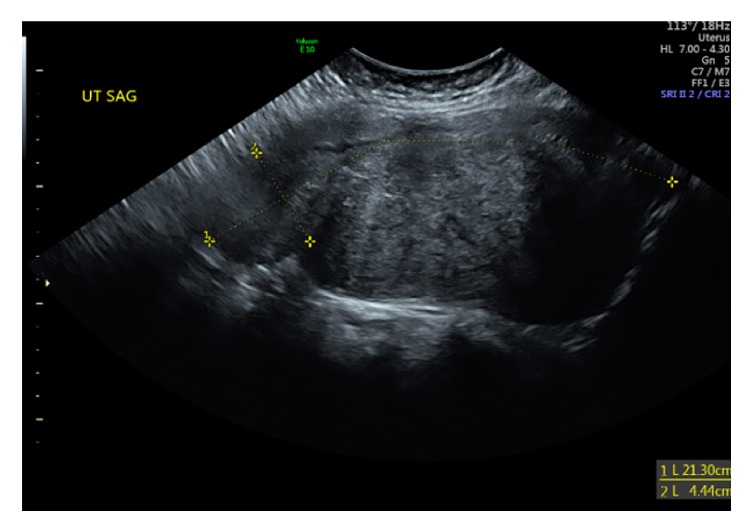
US image of fibroid uterus 6 weeks after treatment.

The patient was admitted to the hospital. Her initial beta-hCG value was 41,542 mIU/mL, and her hemoglobin was 12.7 g/dL. Under transabdominal ultrasound guidance, fetal intracardiac potassium chloride (KCl, 1 mEq/mL) injection achieved cardiac asystole. Upon cessation of fetal cardiac activity, she received methotrexate at a dose of 50 mg/meter squared (body surface area: 1.71 meters squared). The patient tolerated the procedure well and was discharged the following day. On day 4, the patient received the second dose of intramuscular methotrexate (50 mg/meter squared), and beta-hCG was 24,242 mIU/mL. Two weeks later, the patient had an episode of heavy vaginal bleeding and presented to another hospital with hemoglobin of 9.9 g/dL. She was evaluated there and deemed stable. Upon her return to our clinic, she was experiencing bleeding like a menstrual period with stable hemoglobin of 9.9 g/dL. The patient was followed up with weekly beta-hCG values. Five weeks after the procedure, her beta-hCG was negative. Follow-up ultrasound 6 weeks after initial treatment confirmed that were no remaining products of conception in the uterine cavity (Figure 3).

## 3. Discussion

This case demonstrates that conservative medical management with fetal intracardiac KCl injection and intramuscular methotrexate is a safe and effective option for patients in whom uterine aspiration is not possible or is technically challenging. There are no cases in the published literature that demonstrate the use of this method to terminate a pregnancy in a grossly distorted fibroid uterus. This is an appealing option if the patient does not desire surgical management or is a high-risk surgical candidate. It is also safer for a patient who has had multiple cesarean sections and an anatomically distorted uterus as it avoids the risk of uterine rupture associated with the use of misoprostol or mifepristone. The use of KCl and methotrexate has been described in the literature as conservative treatment of nontubal ectopic pregnancies, such as cervical and cesarean-section scar ectopic pregnancies. In one study, patients with cervical ectopic pregnancies were treated with methotrexate alone or methotrexate and KCl [3]. The data showed that KCl and methotrexate were more effective when fetal cardiac activity was seen. The second study demonstrated that this same treatment was effective for nontubal ectopic pregnancies [4].

There are published case reports in which patients with a large fibroid uterus desired termination of pregnancy. Crenin published a case report in which a patient with a twenty-one-centimeter uterus and eighteen total fibroids received methotrexate and 800 mg vaginal misoprostol [5]. This patient returned to the clinic two days later and was no longer pregnant. There is another series of four cases in which patients received methotrexate and vaginal misoprostol, and all had successful terminations without any major morbidity [6]. The case presented here demonstrates another option for medical management using fetal intracardiac KCl and intramuscular methotrexate for first-trimester termination of pregnancy complicated by massive fibroid uterus.
